# Hospital and Institutional News

**Published:** 1917-11-17

**Authors:** 


### HOSPITAL AND INSTITUTIONAL NEWS.

### THE ROYAL FREE'S APPEAL.

DR. MARY SCHARLIEB, in her letter to the *Times* on behalf of the Royal Free Hospital, puts the case in two sentences: "The growing demand for women doctors at home and abroad . . . has resulted in an enormous increase in the number of students. This makes a corresponding addition of hospital space an absolute necessity, for training must be followed by wide practical experience. . . . The school and the hospital cannot be separated." The qualified woman doctor will be needed assuredly in the crisis of reconstruction, as she is still being needed in the crisis of war. The appeal is for £200,000 to make use of the adjoining site, which the hospital owes to the generous foresight of Mr. Alfred Langton, the chairman of the hospital.

### ANTI-RESEARCH PROPAGANDA.

A SHORT pamphlet published by a body styling itself, with voluntarily or involuntarily obscure brevity, "The British Union," may have reached some of our medical readers lately. It is written by Dr. Hadwen and deals with the Registrar-General's recent mortality and other statistics from the point of view of one who thinks that all animal experimentation is wrong, and all results obtained from it worthless. Even granting that this is honest conviction, and waiving for the moment all the great shattering arguments usable against such a position, there remain some quite superficially situated fallacies which are perhaps worth noting here. The author claims that Sclavo's serum is inert in woolsorters' disease, because "the number of deaths in 1915 is double that of fifteen years ago. The average number of deaths per annum during the above period is sixteen. The average number of deaths per annum before the serum was introduced in 1899 was only eight." He does not note the vast increase in the population and in the clothing trade during that period, causing increased handling of wool and hair and, therefore, more chance of anthrax infection; nor the fact that the deaths in 1912 and 1913 averaged hardly more than half the average annual mortality of the previous years quoted by him; nor that in 1915, owing to the war, more wool was handled in the West Riding than ever before. The effect of a rising longevity of the population on the incidence of cancer is ignored; rising longevity being an effect of hygiene, the anti-vivisector's one object of laudation. For a medical man, if he be at all up to date, to write as Dr. Hadwen does of salvarsan without reservation, is, in fact, a *suppressio veri*. We will be more charitable, however, and style the pamphlet merely a crudity.

### MEASLES ON BOARD SHIP.

ADMIRAL CALLIGAN, Admiral of the Fleet, has forwarded to the Southend Corporation correspondence which has passed between the Senior Naval Officer, Southend, and the Chief Constable with reference to the question of the removal to hospital of a case of measles which had occurred on a Norwegian ship lying in the Thames and asking for advice as to the action to be taken in any future similar case. The Town Clerk pointed out that it was not the practice for local authorities to make separate provision for the reception or treatment of cases of measles occurring amongst the civil population; that if such cases were received into the borough sanatorium it would necessitate special accommodation and a special nursing staff; that the lack of accommodation and the difficulty of obtaining nurses, apart from other considerations, precluded the possibility of the adoption of that course; and that, consequently, the Corporation could not undertake to receive cases of measles. However, with regard to cases of infectious disease other than measles, the medical officer of health has been asked to consider the question of receiving a limited number of merchant seamen into the sanatorium.

### AN EPIGRAM ON SALONIKA.

THOSE who read Dr. B. Holroyd Slater's recent article in THE HOSPITAL, entitled "A Hospital Day in Salonika," will not have failed to notice his careful insistence upon the good points of the life there. It is obvious, indeed, that he wished to correct a prevalent dislike of the place, and to provide an antidote to the grumbles in which hospital staffs there, as elsewhere, indulge. We are in a position to supply the grumble itself, which is pithily put in a current saying. "They say here," writes a V.A.D., "that in the first year you lose your hair, in the second year your teeth, and in the third your reputation." The epigram explains Dr. Slater's insistence on the more agreeable side of life in Salonika.

### ANTI-TYPHOID INOCULATION IN FRANCE.

FURTHER proof, if any were needed, of the value of anti-typhoid inoculation is forthcoming in some interesting figures quoted by the Paris correspondent of the *Lancet*, of the case-returns of the French Army for this disease. Inoculation against typhoid was introduced in February 1914, and by August only 125,000 men had been dealt with. The first vaccine used immunised solely against Eberth's bacillus, and it was not until later that vaccines were introduced against paratyphoid infection A and B, the significance of which was only slowly recognised. In the twelve months, November 1, 1914, to October 31, 1915, 87,618 cases with a mortality of 8,614, or 9.8 per cent., were recorded, whilst taking the two periods of six months, that ending April 30, 1915, shows 58,014 cases with 7,013 deaths (15.3 per cent.), and the second period, May 1 to October 31, 29,604 cases with 1,151 (3.9 per cent.) mortality. The number of cases was thus reduced during the second period to approximately one-half, and the mortality per thousand men became seven times less, owing to the more general use of the vaccine and the better provision of pure drinking-water. Vaccination against paratyphoid was introduced in November 1915, and in the next following six months the number of cases fell to 12,043, and the deaths to 302 (2.5 per cent.); from May 1, 1916, to October 30, 1916, the case rate was still further reduced, and finally, from November 1, 1916, to September 1, 1917, the number of cases fell to 2,048 and 129 deaths. It has been calculated that if the frequency and mortality of typhoid had remained what they were at the end of 1914, the number of cases would have exceeded a million, and the resulting deaths 145,000, without taking into account the probable aggravation of the disease caused by long-continued fighting in the same areas. There is no doubt, however, that improved sanitary conditions have had an important share in procuring this great reduction.

### A SAILORS' FUND SUNDAY?

A PUBLIC-SPIRITED journalist can hardly devote too much space to King George's Fund for Sailors. We believe that the public wishes to be much better informed of the doings of the sailors and the Mercantile Marine than it is at present, and, further, that a mass of interesting information which could be of no use to the enemy is being inconsiderately withheld. Public ignorance on this head is not the public's fault; but, fortunately, the censorship does not apply to the doings of the Fund which King George has permitted to be founded for the benefit of the seamen and their dependants. Last week's meeting, of which a report appears, is full of interest to hospital people. It quotes the total already subscribed. It gives the percentage of cost incurred in raising the money. It makes a novel suggestion for increasing the income of the Fund. For the former figures our readers are referred to the report. The latter suggestion may here be noted. It was made by Sir Richard Williams-Bulkeley, who presented the report of the Collecting Committee. He remarked that the co-operation of the clergy was growing, and that the lead given by the Archbishop of Canterbury, in the letter which we are glad to print, " would no doubt result in a special Sunday being set apart for collections on behalf of King George's Fund for Sailors."

### A DANGEROUS COMPETITOR?

THE above is a revolutionary proposal. It may be held to create a precedent. It may be regarded as a dangerous competitor to Hospital Sunday, especially in those places where Hospital Sunday has shown a decline. We are therefore bound to become its sternest critics; and yet we are not sure that Sir Richard Williams-Bulkeley was ill-advised to make it. At all events, we will go so far as this. We will say that if another Sunday is to be devoted to church collections for charity other than that of the voluntary hospitals, we can think of no claim, compared to theirs, of equal importance for such a service, unless it be that of King George's Fund for Sailors. That is, let us admit, a very large concession. It is a concession made to a supremely patriotic cause. The cause alone makes the idea of another "Hospital Sunday" even thinkable. No decision can be made by the Archbishops until the clergy and the hospitals have been consulted; but for the sake of England's seamen we think that they should be consulted on this radical, and suggested, change.

### SHOULD THE RED CROSS SUPPLEMENT THE GOVERNMENT GRANT?

AN interesting phrase was dropped by the Marquis of Bristol in presiding at a special meeting which had been called to consider the best means of meeting the deficit on the year's working of the West Suffolk Hospital. Apart from the increase in the cost of provisions and appliances, he declared that one reason for the deficit was that the Government allowance for wounded soldiers was not nearly equal to the present cost. He then made the interesting statement that last year there would have been difficulties if the Red Cross Society had not given a donation of £350 "towards the extra cost of the wounded soldiers." If these are the *ipsissima verba* which accompanied the donation, they provide a capital illustration of the present claim that the Government grant is insufficient even for maintenance. No suggestion is made here of the desirability of increasing the grant so as to pay the hospital rent and interest No suggestion is made here that professional attendance should be paid for. It is a straight issue. At Bury St. Edmunds, at all events, the Red Cross has given to the West Suffolk Hospital a definite grant towards a definite deficiency. Since the grant was designed to pay for the cost of maintaining the wounded, since the Red Cross has publicly supplemented that grant, here is the final proof, surely, that the time has come for the grant to be raised.

### SECTIONAL SUBSCRIPTIONS IN GLASGOW.

LAST March the Royal, the Western, and the Victoria Infirmaries of Glasgow issued a special and joint appeal in the hope of being able to raise by its means sufficient money to tide them over the rise in prices. Apparently the aim was to raise sufficient not so much to reduce old debts as to meet the increased cost of annual maintenance. The position now is as follows: The deficit on the ordinary account of the Royal Infirmary for the year 1916 was £30,000; on the Western, £22,000; the deficit on the Victoria Infirmary was smaller. Because of the appeal the ordinary subscriptions of the Royal (within two months of its financial year) have increased by £5,400, of the Western by £2,000, and of the Victoria by some £1,600. We learn that even so extraordinary revenue will have to be realised to the same extent as last year. Therefore, in effect, the position has been prevented from getting worse—an important negative gain; but it has not been made better. But when we remember the enormous industrial expansion of the Glasgow area, the increase of £5,000 from the workmen's subscriptions is, bluntly, a trifle; for it does not represent a tithe of what might have been expected from a thorough organisation of industrial subscriptions. It is, in effect, a small subscription from the people employed in the warehouses and manifold public works of the neighbourhood. A sum of £15,000 from this source alone should be, in fairness and modesty, expected. The only remedy is to complete the organisation by the organisation of sectional subscriptions from every class of worker and resident in the area served by the three infirmaries. Beginnings have been made by Dr. Basil Rhodes at North Staffordshire, and at Cardiff, and at York. Will their example be followed by Glasgow?

### WAR SERVICE OF HEALTH OFFICIALS.

It has been suggested in various quarters that the Public Health Service has been and is still detaining men who would be more usefully employed in the R.A.M.C. The fallacy of these assertions has frequently been pointed out in these columns, and yet further proof lies to hand in the annual report of Dr. A. Maxwell Williamson, medical officer of health for the city of Edinburgh. He records that the energies of his depleted staff were taxed to the utmost, not only with the investigation and prevention of disease among the civilian population, but also and very specially in supplying the needs of the large number of naval and military men in various centres of the city, and even extending so far as Queensferry. Such work included the taking of swabs from infectious cases and from suspected "contacts," the disinfection of premises occupied by the military, and the investigation of the origin of outbreaks of infectious diseases. Since the commencement of the war over 1,750 such cases have been inquired into by the Edinburgh Health Department, and some 1,600 of these have been removed to hospital. In addition to this, nearly two hundred thousand articles of uniform, bedding, clothing, etc., have been disinfected at the central station, after a full inquiry had been made into the nature of the disease, and the necessity for such a procedure. The work of the medical officer at home, whether military or civilian, is every whit as necessary to the prosecution of the war as that of the surgeon or physician more directly concerned with the welfare of the sick and wounded. These men perform very arduous work year in and year out with little recognition of their services, and it will be only when the full history of the war comes to be written that the tremendous part they have played in investigating and stamping out disease will be adequately acknowledged.

### BRITISH RED CROSS IN ITALY.

We are glad to learn that twelve of the nurses and six men of the unit of the British Red Cross at work under the leadership of Mr. George Trevelyan have reached England in safety from their hospital at Villa Trento, close to the Italian frontier. Established in September 1915, as a mark of the sympathy existing between Great Britain and Italy, they have up till now treated no fewer than 8,000 out-patients and 5,000 in-patients. When the Italian retreat began, the patients were evacuated from the hospitals of the district, but the staff itself did not leave till October 27. The complete *personnel*, consisting of two doctors, twenty-five nurses, five officers, and sixty-five male drivers and mechanics, was saved, but owing to the congested state of the roads near Udine nineteen of the forty-five cars had to be abandoned. A further contingent of the unit is now on its way to England, but we hope that the check is but a temporary one, and that the hospital will be able to resume its useful work with our Allies in the near future.

### A MILLIONAIRE'S MANSION FOR WOUNDED AMERICANS.

Hundreds of palatial private homes, according to the Washington correspondent of the *New York Herald*, in America, France, England, and Canada have been offered to the War Department for the reception of wounded American soldiers. Mr. Vincent Astor, popularly reported to be America's richest young man, has just offered his country mansion on the banks of the Hudson River for the reception of convalescent soldiers returned from France. The estate is estimated to be worth £1,600,000, and is considered the most beautiful of the many fine estates on the river-side. The offer has been made contingent to the house meeting certain requirements for which it is to be examined by the Sanitary Corps of the Army Medical Department. Mr. Astor himself is serving as ensign upon his own yacht the *Noma*, which he placed at the disposal of the British Government at the beginning of the war. None of the other mansions have as yet been accepted, but the American War Office is keeping a list of them, and should the number of wounded arriving from France exceed the limits of accommodation in the military hospitals, the offers will be gratefully accepted.

### A PLAGUE OF PENSION COMMITTEES.

Whatever the merits or demerits of the much-discussed treatment in hospitals of discharged soldiers, there can be no doubt as to the desirability of issuing clear and consistent instructions to Local Pensions Committees. Yet hospital secretaries who have had dealings with these committees find that one local body has an idea of its powers quite different from that of another, while a third has a conception different from both. The Ministry of Pensions has, indeed, issued a pamphlet of instructions which appear to be perfectly definite. Yet when a secretary, told by a Pension Committee that they could not pay more than 7s. a week for a pensioner in-patient, produced the plain statement in the pamphlet that 28s. per week could be paid, he was met with two objections: firstly, that those instructions had probably been superseded by others issued later, and secondly, that that particular committee was only a sub-committee and was acting under the directions of the So-and-So Local Committee! The same secretary has had suggested to him by various committees the sums of 6d., 1s. 6d., 2s. 6d., and 5s., each being declared to be the proper amount to be paid per out-patient attendance.

### THE SUGAR-VOUCHERS AND HOSPITALS.

It was hardly to be expected that a bureaucratic innovation such as the issue of sugar-cards should be launched without a hitch; but it is regrettable that hospitals should have to suffer in connection with the supply of this necessary commodity, through no fault of their own. In Westminster, the Food Control Office was so overworked that the vouchers, which should have been issued to institutions (including hospitals) by October 9, had not been received by them on November 6. In consequence, the retailers have refused to supply sugar to more than one hospital in Westminster, on the ground that the vouchers have not been transmitted to them. Such conduct is, of course, absolutely unjustifiable, as the rationing of institutions does not come into operation (according to the plan first laid down by the Controller) until November 9. In one instance, where a large department store refused to supply a hospital during the third week of October, the secretary reported the matter to the Food Office, and was informed that if there were a continuance of such conduct, the retailer stood in danger of having his whole supply of sugar cut off. The secretary forwarded this information to the store concerned, and immediately received a consignment of sugar—though even then there was sufficient only for the patients, and the staff have had to do without sugar for more than a fortnight.

### AMERICAN WOMEN DOCTORS.

It is stated that 4,000 American medical women have been asked to register their names for service in ten American women's hospitals. Some of these will commence their war service in the Women's Army General Hospital, the management of which is entirely in the hands of women, and we are told that the funds have been raised by women doctors. It is said that one unit of American women doctors intends to open hospitals in the devastated regions of France and Belgium, and these hospitals are for women and children. We give the number of American medical women as reported in the press, but we are inclined to think that in the number 4,000 it is not unlikely that the numbers of medical women and nurses have been lumped together. It is interesting to learn that both nurses and doctors are to have lessons in French, so that they can talk to their patients, and at the conclusion of these lessons they are to have an examination at a French hospital. It is certainly highly important that the ordinary medical phrases should be learned by all who have to look after sick foreigners, for the ordinary phrase-books do not contain what is wanted.

### WHY BLANKET RATIONING IS IMPERATIVE.

Some facts about the blanket organisation scheme are given in a letter which the Director of Raw Materials has sent to the Hull Corporation. The communication observes that, from the return sent in as to blanket requirements and the stocks held, the Corporation possesses a number of blankets which are not likely to be brought into use during the ensuing winter and points out that there are many authorities which are in want of blankets. In view of the paramount necessity for minimising the demands upon wool and upon the blanket-making machinery of the country, which is now organised for naval and military purposes, every endeavour should be made to utilise all available blankets. The Director of Raw Materials therefore intimates that those authorities who need blankets have been informed of the available stocks of other authorities, and if they are unable to obtain the blankets they need from the existing stock of a manufacturer, agent, or retailer, it is hoped that they may be able to procure them from the stock of a neighbouring authority, if the blankets are at all suitable. The Local Government Board approves of this proposal. The Director, therefore, has now requested the co-operation of the Hull Corporation in this scheme, explaining that it is solely necessitated by the present emergency. In the event of an existing surplus stock being disposed of and an unforeseen demand arising later, the War Office Contracts Department will assist in the provision of additional blankets. The Corporation Health Committee has accordingly promised to give sympathetic consideration to requests which may be received from any neighbouring authority.

### MEMORIAL TO DR. W. A. HASLAM.

The story of a young medical man's self-sacrifice was told at a ceremony of an unusual character which took place at the Hull Sanatorium on November 7, when the Lord Mayor, in the presence of members of the Hull Health Committee and representatives of other public bodies, unveiled a bronze tablet in memory of Mr. W. A. Haslam, M.R.C.S., L.R.C.P., medical officer of the Corporation isolation hospitals, who died a year ago from scarlet fever caught while attending his patients. It was hoped to have the presence of Mr. Haslam's mother and sister, who reside in Stroud Green, London, but a letter was read thanking the Corporation for the kindness shown and regretting their inability to attend the unveiling of the memorial. Alderman Askew, in uncovering the tablet, paid a notable tribute to Mr. Haslam's zeal, adding that "he laid down his life for his patients. His patients regarded him with affection and he was devoted to them." Mr. Watson Boyes, deputy-chairman of the Health Committee, said they would always remember Mr. Haslam as the friend of all classes of patients committed to his care. It may be noted that the Hull Sanatorium, which ranks as one of the finest of its kind in the country, will also remain as a memorial of Mr. Haslam's work, for he took a large share in planning this institution for the treatment of tuberculosis.

### A BELOVED HOSPITAL COMMANDANT.

The soldiers who have passed through the Royal Pavilion Hospital and its annexe at York Place

School, Brighton—and they are many—will remember the Commandant, Colonel Sir Neil Campbell, and will hear with general regret that he is retiring from his position as chief of the medical staff. The dusky warriors from India will remember him: how nothing was left undone to meet many of their wishes and fancies peculiar to their caste. The limbless "Tommy," also, for it is within the confines of the hospital that one of Queen Mary's Workshops has been established, and those who have already passed through the shops and are now in civilian life helping themselves as citizens as far as their circumstances will allow, will never forget "The Colonel." His work as Commandant of one of the largest military hospitals in the country has been no sinecure, but he always set a splendid example in all his professional duties, and his personal qualities were most engaging. His work for the "boys" has been a labour of love, blending as he always has done the elements of modesty, sweetness, and simplicity with all the knowledge that he possessed in medicine and surgery, on behalf of those who have made such sacrifices for their King and country. On more than one occasion Colonel Sir Neil Campbell has had the honour of receiving both the King and Queen at the Brighton Hospital, besides other members of the Royal Family, the late Lord Kitchener, and many other important personages both in a private and official capacity.

### A PROUD FAMILY RECORD.

The death of Mrs. T. Fielding Johnson removes from the public life of Leicester a lady who has long been associated with the institutional life of the town, and whose influence was always exerted in the promotion of the welfare of the community. Mrs. Fielding Johnson was for years a member of the board of governors and of the house committee of the Royal Infirmary. She was the first lady to undertake administrative work for the institution, and in this capacity her advice and counsel were of great value, and her service marked by many acts of helpfulness and generosity. She was deeply interested in, and closely associated with the progress and development of Sir Edward Wood's reconstruction scheme. The Queen Victoria Memorial Wing and the Nurses' Home were formally opened by her, and the naming of the "Fielding Johnson" ward in the new Children's Hospital after her and her bereaved husband perpetuates the names of two of the institution's great benefactors. She was one of the founders of the local Maternity Hospital, which has proved a boon to poor women, and she was greatly interested in the "Faire" Hospital, which provides hospital treatment for those of limited means who cannot provide for treatment in nursing homes. She also took a great interest in the educational life of the town, and was a member of the original board of managers of the well-known Wyggeston Girls' School. In 1891 she published in "Glimpses of Ancient Leicester" a comprehensive history of the town and its antiquities, on which she was a recognised authority. Mr. and Mrs. Fielding Johnson celebrated their golden wedding in 1913. Mr. Fielding Johnson, the survivor, who is in his eighty-ninth year, is the senior vice-president and the oldest governor of the infirmary. He succeeded the late Sir Arthur G. Hazlerigg, Bart., as chairman of the weekly board of the institution, in which capacity he rendered memorable service at an important period. He was a member of this board for the long period of forty-one years. It was a source of much satisfaction to him when in 1915 his son, Mr. T. Fielding Johnson, jun., was elected to the chairmanship to continue a family association which has been of inestimable value to the infirmary.

### EDUCATIVE REST CLUBS FOR SOLDIERS AND NURSES.

The Birmingham system of rest-rooms for wounded in the city has led to the opening of a large additional rest-room at Daimler House, Paradise Street, once a show-room of the Daimler Motor Company. The room has been furnished by the Birmingham furnishing trades free of charge, and is capable of accommodating 1,000 wounded a day. Arrangements are being made for the men to be taught light trades, and a motor chassis is being displayed, so that the men can pick up some idea of motor work if they wish it. Also a special department is being opened at the Daimler House for trained nurses to meet in a rest-room, and a portion is being allotted for married wounded men who may have their wives or relatives coming from a distance to visit them, so that they can meet at this room and have a rest and refreshments. Here, too, wounded coming from country hospitals to the Birmingham theatres can have tea before leaving the city. In Birmingham these arrangements have gone hand in hand with the organisation on a big scale of entertainments for the wounded. We described the scheme at the time of its inception and are glad to report its successful growth.

### THE TREATMENT OF VENEREAL DISEASES IN NOTTINGHAMSHIRE.

Complaints were made at a meeting of the Nottingham County Council on October 30 that the holding up of the scheme for the establishment of a treatment centre for venereal diseases at the Nottingham General Hospital was due to the difficulties raised by the Local Government Board. Mr. C. A. Houfton, in presenting the report of the Public Health Committee, observed that if only the Local Government Board would help them they would be able soon to make a start with the treatment of venereal diseases. At Mansfield it is proposed to acquire for this purpose suitable houses adjacent to the hospital. It was also reported to the County Council that maternity and infant welfare centres have been established at Carlton and Netherfield and at Stapleford. The difficulties in regard to adequate premises for the tuberculosis dispensary at Newark have not yet been settled.

### A DISPUTED POINT.

A novel point was raised at a meeting of the Cumberland County Council at Carlisle on November 7. The Health Committee recommended that the chairman of the County Midwives Committee, who is Lady Mabel Howard, be invited to attend the meetings of the Health Committee, and be allowed to take part in the discussion of matters appertaining to her sphere of work, but not to vote. On the question of the principle involved—viz., that the chairman of the Midwives Committee was not a member of the County Council—a member of the Council, Mr. Jefferson, objected to this minute and moved its deletion. Fifteen other members agreed with him, but thirty-six members supported the Health Committee on the division.

### THE PRINCE OF WALES' HOSPITAL, CARDIFF.

Some time ago, a request was made to the Council of the National Relief Fund for a grant to be made towards the work of the Prince of Wales' Hospital for Limbless Sailors and Soldiers, Cardiff, and in response to this request a grant of £5,000 has now been made from the Fund. It is pleasing to know that the good work of the Prince of Wales' Hospital is thus officially recognised, and that the investigations made by the Council of the National Relief Fund before making the grant have proved satisfactory.

### THE "FRANCONIA" NURSING SISTERS.

Some interesting details regarding the history and growth of the Canadian Nursing Service were forthcoming at a dinner which was held recently at the Criterion to mark the anniversary of the arrival in this country of the first contingent of Canadian nursing sisters. Matron Smith, of the Ontario Military Hospital, presided over the little gathering of forty nurses, all members of the original contingent which came to England just over three years ago. The guest of the evening, Matron-in-Chief Margaret Macdonald, told the story of the "*Franconia* contingent of the C.A.M.C.," so called because that was the name of the liner in which they crossed the Atlantic. The contingent comprised two matrons and 100 nursing sisters. Of that number one had since died, twelve had been married (and two more are to be married at Christmas), four had resigned, six had been reported permanently unfit for active service (and were now on duty in Canada), twelve were on transport duty and sick leave, and the remainder on duty in England and France. Ten of the sisters in the original contingent had been promoted to matrons, and five were acting-matrons. Canadian nursing sisters had seen service, under fire in some instances, in England, France, Egypt, Lemnos, Salonica, and Russia. The Service had grown, until now it was composed of one Matron-in-Chief, twenty-five matrons, eleven acting-matrons, and 1,683 nursing sisters. The first gallant hundred shared between them many honours, their remarkably fine record being:—Thirty-nine Royal Red Crosses, one Victoria Medal, and two Médailles des Epidémies. They may well be proud of their achievements.

### THE MATRON-INSPECTOR.

The suggestion lately made in The Hospital that ex-matrons might make better inspectors of military hospitals than the men who now, often perfunctorily, discharge the task, has evoked one humorous criticism. It is urged that there is much in the suggestion, but that the success of the plan, if adopted, would depend upon no friendship existing between the inspector and the matron of the hospital inspected. We ourselves should hardly have ventured to suggest that women were more open to private influences than men in regard to their public work, but this brief is held by the person, a woman, who urges it. Matrons, of course, make friends among nurses of their own rank. They also are open to the temptations which beset average human nature. While admitting this, however, we do not think that the risk of favouritism need make ex-matrons ineligible for a hospital inspector's work. Their other qualifications are too numerous. Is it too much to hope that the Director of the Army Medical Service may consider the suggestion and act upon it?

### THIS WEEK'S DRUG MARKET.

The upward tendency of drug prices continues, although there are few actual changes in value of particular moment to report. It is expected that before next week makers of iodides will raise their quotations for potassium iodide, sodium iodide, and iodoform. Saccharin is more plentiful and prices are rather lower. Salicylates continue to be in fairly good supply, and prices are unchanged. There is no change in the prices of bromides, but ammonium bromide is in rather short supply. Castor oil of medicinal quality is extremely scarce. Phenacetin is again slightly lower in price. Cocaine is still scarce, and business has been done at a very high rate. The upward tendency in the price of honey continues. Liquid paraffin is in more plentiful supply, and the price has a rather lower tendency; hard paraffin is also more plentiful and somewhat cheaper. Exceedingly high prices are paid for any olive oil that may be available. Antifebrin still tends upwards in price.

### TO OUR READERS.

Contributions are specially invited from any of the readers of The Hospital. They should deal with topical subjects and news or incidents illustrating any department of hospital work, its progress or difficulties. They must be authenticated for the information of the Editor only. The minimum payment if published is 5s. There is no hard-and-fast rule as to space, but paragraphs of about twenty lines in length are preferred.

